# Prostate-Specific Membrane Antigen (PSMA) Expression in Tumor-Associated Neovasculature Is an Independent Prognostic Marker in Patients with Ovarian Cancer

**DOI:** 10.3390/jpm12040551

**Published:** 2022-03-31

**Authors:** Gerda Hofstetter, Christina Grech, Dietmar Pils, Johannes Pammer, Barbara Neudert, Nina Pötsch, Pascal Baltzer, Tatjana Traub-Weidinger, Veronika Seebacher, Stefanie Aust

**Affiliations:** 1 Department of Pathology, Comprehensive Cancer Center (CCC) Vienna, Medical University Vienna, Waehringer Guertel 18-20, A-1090 Vienna, Austria; gerda.hofstetter@meduniwien.ac.at (G.H.); johannes.pammer@meduniwien.ac.at (J.P.); barbara.neudert@meduniwien.ac.at (B.N.); 2 Department of Obstetrics and Gynecology, Comprehensive Cancer Center (CCC) Vienna, Medical University Vienna, Waehringer Guertel 18-20, A-1090 Vienna, Austria; christina.grech@meduniwien.ac.at (C.G.); stefanie.aust@meduniwien.ac.at (S.A.); 3 Division of Visceral Surgery, Department of General Surgery, Comprehensive Cancer Center (CCC) Vienna, Medical University Vienna, Waehringer Guertel 18-20, A-1090 Vienna, Austria; dietmar.pils@muv.ac.at; 4 Department of Radiology and Nuclear Medicine, Medical University Vienna, Waehringer Guertel 18-20, A-1090 Vienna, Austria; nina.poetsch@meduniwien.ac.at (N.P.); pascal.baltzer@meduniwien.ac.at (P.B.); tatjana.traub-weidinger@meduniwien.ac.at (T.T.-W.)

**Keywords:** PSMA, neovasculature, ovarian cancer

## Abstract

Prostate-specific membrane antigen (PSMA) is present in the tumor-associated neovasculature of many cancer types. Current data in ovarian cancer are limited and controversial; thus, the aim of this study was to investigate PSMA expression in a larger and homogenous patient cohort. This might lead to further studies investigating the use of imaging and therapeutic modalities targeting PSMA. Eighty patients with advanced stage high-grade serous ovarian cancers were included. Using immunohistochemistry, PSMA and CD31, a marker for endothelial cells, were examined in whole tissue sections. Percentage and intensity of PSMA expression were determined in the neovasculature. Expression levels were correlated with clinicopathological parameters and survival. Low (≤10%), medium (20–80%), and high (≥90%) PSMA expression was found in 14, 46, and 20 ovarian cancer samples, respectively. PSMA expression was confined to tumor-associated neovasculature and significantly correlated with progression-free (HR 2.24, 95% CI 1.32–3.82, *p* = 0.003) and overall survival (HR 2.73, 95% CI 1.41–5.29, *p* = 0.003) in multivariate models, considering age, FIGO stage, and residual disease. This is the first study showing a clinical relevance for PSMA in patients with ovarian cancer. PSMA was detected in the vast majority of cancer samples and showed an impact on survival.

## 1. Introduction

Neovascularization is a hallmark of carcinogenesis. It provides cancer cells with oxygen and nutrient supply, thus allowing their survival and rapid growth [1]. Tumor-associated vessels differ from regular vasculature. Prostate-specific membrane antigen (PSMA) has been exclusively detected in the vascular endothelium of different cancer types such as oral squamous cell, thyroid, breast, hepatocellular, and renal carcinomas as well as gliomas, but not normal vessels [2,3,4,5,6,7].

PSMA is a type II transmembrane protein that was initially found on the surface of normal and malignant prostate cells [8]. While a ligand has not been identified yet, PSMA has peptidase activity that might suggest a role in signal transduction and nutrient uptake, related to glutamate and folate absorption, respectively [9]. Imaging tools targeting PSMA have been developed and evaluated in large clinical trials [10]. Ga-68 PSMA-11 and piflufolastat F-18 PET seem to possess improved sensitivity and specificity for the detection of lymph node and distant metastases compared to conventional imaging. Both were recently approved for patients with prostate cancer by the FDA. In addition, PSMA can be targeted therapeutically with minimal adverse events. 177-Lu-PSMA-617 shows promising response rates in patients with metastatic prostate cancer [11]. First experiences with PSMA-directed imaging and treatment modalities have also been reported in patients with thyroid, breast, hepatocellular, renal, and lung cancer as well as glioblastoma [12]. To evaluate if patients with epithelial ovarian cancer (EOC) may benefit from these innovative techniques, we focused on PSMA expression patterns in EOC tumor tissue. Current data on PSMA expression in ovarian cancer are controversial and limited. While one study found PSMA in the tumor-associated neovasculature of all cases, PSMA was absent in all samples in another study [13,14]. Of note, both studies comprised small collectives of 21 and 32 patients.

The aim of the present study was thus to examine PSMA expression patterns in a homogenous group of patients with advanced high-grade serous ovarian cancer (HGSOC) and evaluate its clinical relevance and impact on outcome.

## 2. Materials and Methods

### 2.1. Study Population

All patients treated for EOC at the Department of Gynecology, Medical University Vienna, between 2010 and 2018 were identified from a prospectively maintained database. Inclusion criteria consisted of advanced stage (FIGO III-IV), high-grade serous histology, available chemo-naïve representative tumor tissue from primary debulking surgery or diagnostic laparoscopy at the time of diagnosis and respective clinical and follow-up data. Clinical and follow-up data were derived from electronic medical records. Patients were treated according to local standards and international guidelines with primary or interval debulking surgery and platinum-based chemotherapy. Bevacizumab was added at the discretion of the attending physician after exclusion of contraindications. Overall observation time was the time interval between diagnosis and last contact. Patients without recurrence, cancer progression, or death were censored at the time of last medical follow-up in case of progression-free survival or last contact for overall survival. Experienced gynecological oncologists and pathologists performed the clinical and histopathological evaluation and determined response to first-line treatment.

### 2.2. Immunohistochemistry

Representative tumor tissue blocks were selected. In patients treated with neoadjuvant chemotherapy, tissue obtained at pretherapeutic diagnostic laparoscopy was used.

Immunostaining was performed on 2 µm thin whole tissue sections with a Benchmark Ultra autostainer (Ventana Medical Systems, Tucson, AZ, USA). For PSMA the antibody AC-1060 (Epitomics, Burlingame, CA, USA) and for CD31 the antibody M823 (Dako, Agilent Santa Clara, CA, USA) was used at dilutions of 1:50 and 1:20, respectively. Immunostaining was carried out following in-house validated protocols [15]. Briefly, slides containing unstained sections were pretreated performing heat-induced epitope retrieval in cell conditioning 1 (CC1) buffer (heating temperature 95 °C), pH 8 (Ventana Medical Systems), for 64 min. Incubation times for primary antibody were 44 and 60 min at 36 °C. For visualization of antigen–antibody binding, the ultraView Universal DAB detection kit (Ventana/Roche) was used, which contains a cocktail of horseradish peroxidase (HRP)-labeled antibodies (goat anti-mouse IgG, goat anti-mouse IgM, goat anti-rabbit).

Slides were independently evaluated by two dedicated gynecological pathologists (G.H. and J.P.). CD 31 was used to identify tumor-associated vasculature. Vessel density in the tumor stroma was estimated using three categories (low, medium, and high) and then used as a reference for scoring PSMA positivity. PSMA expression was determined semiquantitatively as the percentage of vessels that were positive for PSMA (0–100%, in steps of 10). All vessels present in a whole tissue slide were evaluated for their PSMA expression. Staining intensity was evaluated by a 4-tier grading system (negative, weakly positive, moderately positive, and strongly positive).

### 2.3. Data Analysis and Statistics

Statistical analyses were performed with R and R packages survival v3.2–12, pspline v1.0–18, and MASS 7.3–54 [16,17,18]. The association of the PSMA expression (0–100%, in steps of 10) with censored overall survival outcome data was modeled by a non-linear Cox regression method. Using smoothing splines, a penalized spline basis was specified for the PSMA predictor, and the PSMA-dependent hazard ratio (HR) was plotted against the PSMA expression with its 95% confidence intervals. Cutoffs were set at the crossings of the HR estimate with the HR = 1 line, i.e., ≤10% and ≥90%. Thus, patients with low and high PSMA expression were combined to an unfavorable survival group and compared to the PSMA medium expressing group as favorable survival group. Finally, this dichotomous PSMA predictor was used in univariate and multiple Cox regression analyses for progression-free and overall survival. For the presented multiple Cox regression models, age, FIGO stage, and residual tumor mass after debulking surgery were used together with the dichotomous PSMA-predictor (“low/high” versus “medium”) and the final model dichotomized and shown as survival curves. As these survival curves represent a multiple Cox regression model, no censored patients are indicated.

## 3. Results

### 3.1. Study Population

A total of 80 patients with HGSOC were included in the study. Median patient age was 60.4 years (range, 33.3–81.8). All patients had advanced disease; 59 patients presented with FIGO stage III and 21 with FIGO stage IV. Primary debulking surgery was performed in 59, and neoadjuvant chemotherapy was administered in 21 patients. Complete gross resection was achieved in 43, while visible residual disease remained in 37 patients. All patients received platinum-based chemotherapy. Bevacizumab was added in 64 (80%). After six cycles of chemotherapy, complete serological and radiological response was observed in 68% (*n* = 49) patients. One patient declined chemotherapy, and seven were lost to follow-up. Time to first progression ranged from 5.7 to 76.3 months. During the median follow-up of 56.6 (range, 44.2–70.3) months, 64 recurrences and 42 deaths were observed.

### 3.2. Immunohistochemistry

**Figure 1:**
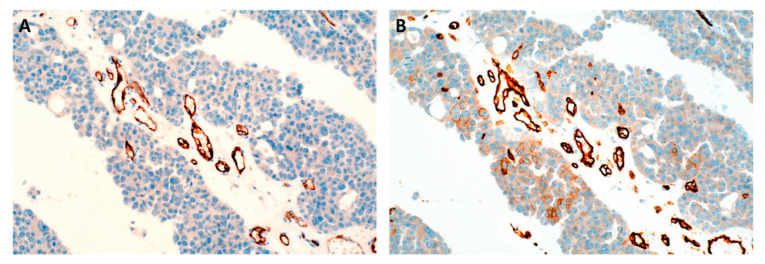
Representative immunohistochemical images of ovarian cancer samples. On the left, 90% tumor-associated vessels exhibit PSMA staining (**A**). On the right, vessels are delineated using a CD31 antibody (**B**); 20× magnification.

As limited data are available on PSMA expression patterns in EOC tissue, we performed IHC on whole tissue sections (Figure 1). We focused on PSMA expression in neovasculature of primary EOC tissue. Cumulative PSMA expression was evaluated in the entire cancer tissue and was not restricted to selected foci with highest vessel density. Tumor-associated neovasculature is not evenly distributed; thus, whole tissue slides might be advantageous compared to small cancer sections analyzed in tissue microarrays. Membranous and cytoplasmatic staining was observed. Expression rates varied between 0% and up to 90%. Percentages were distributed as follows: 0% (*n* = 8), 10% (*n* = 6), 20% (*n* = 3), 30% (*n* = 6), 40% (*n* = 7), 50% (*n* = 1), 60% (*n* = 14), 70% (*n* = 5), 80% (*n* = 10), 90% (*n* = 20), and 100% (*n* = 0). Staining intensity was classified as negative (*n* = 8), low (*n* = 8), medium (*n* = 29), and high (*n* = 35). As staining intensity significantly correlated with the percentage of positive cells (Spearman′s rank correlation coefficient ρ = 0.72), it was not included in further analyses. Staining intensity also has limited reproducibility as it is influenced by factors such as the fixation method, storage time of unstained tissue slide, and variations in immunohistochemical protocols [19]. Thus, scores using expression percentages are preferred in clinical practice. PSMA expression was confined to tumor-associated neovasculature, sparing adjacent regular vessels. Of note, ovarian cancer cells lacked PSMA expression in all cases.

### 3.3. Survival Analyses

**Figure 2:**
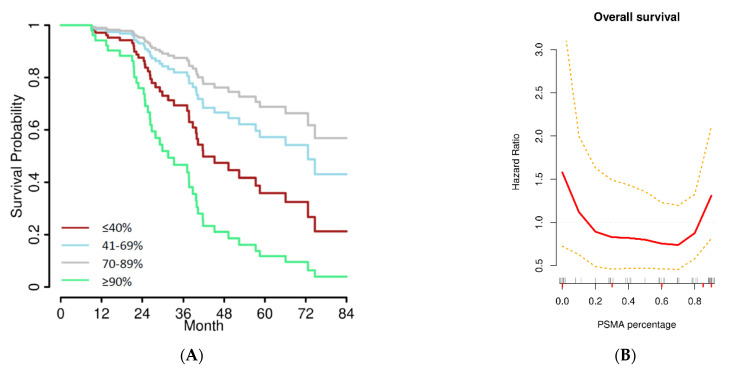
(**A**) Overall survival stratified by quartiles of PSMA expression in a multivariate model considering age, FIGO stage, and residual tumor. Using a univariate Cox regression, the distribution of median overall survival for the four groups was as follows: 66.1 months (70–89%), 57.3 months (41–69%), 39.9 months (≤40%), and 38.8 months (≥90%). As these survival curves in (A) represent a multiple Cox regression model, no censored patients are indicated. (**B**) Hazard ratio for overall survival significantly increased in cases with low (≤10%) and high (≥90%) PSMA expression (crossing the red HR curve with the HR = 1 line in grey); thus, they were combined for further analyses (“low/high” PSMA group, *n* = 34) and compared to cases exhibiting PSMA expression between 20% and 80% (“medium” PSMA group, *n* = 46). Small bars at the top of the *x*-axis indicate single samples (jittered), and red bars at the bottom of the *x*-axis are the minimum, the 25th, the 50th, and the 75th percentiles, and the maximum of PSMA expression, the so-called five-number summary.

First, cases were stratified according to quartiles of PSMA expression (≤40%, *n* = 23; 41–69%, *n* = 22; 70–89%, *n* = 15; and ≥90%, *n* = 20). Compared to low PSMA expression, cases with medium PSMA expression (second and third quartiles) showed improved overall survival with median 66.1 (HR 0.79 (95% CI 0.36–1.74)) and 57.3 months (HR 0.43 (95% CI 0.13–1.32)). High PSMA expression (≥90%) was associated with impaired overall survival (median 38.8 months, HR 1.32 (95% CI 0.60–2.91)), similar to the group with low PSMA expression. The same distribution was observed in multivariate analyses considering age, FIGO stage, and residual tumor burden (Figure 2A).

Following the bimodal overall survival risks shown in Figure 2B, we determined the optimal cutoffs by non-linear modelling of the PSMA expression dependent hazard ratio (HR) for further correlation with clinical and survival data (details given in the Methods section), yielding a two-tailed graph, and cutoffs were therefore set at the crossings of the HR estimate with the HR = 1 line, i.e., ≤10% and ≥90%.

Consequently, cases with low (≤10%) and high (≥90%) PSMA were combined (“low/high” PSMA, *n* = 34) and compared with cases exhibiting PSMA expression in the 20–80% range (“medium” PSMA, *n* = 46). PSMA expression was not associated with standard clinicopathological parameters (Table 1).

**Figure 3:**
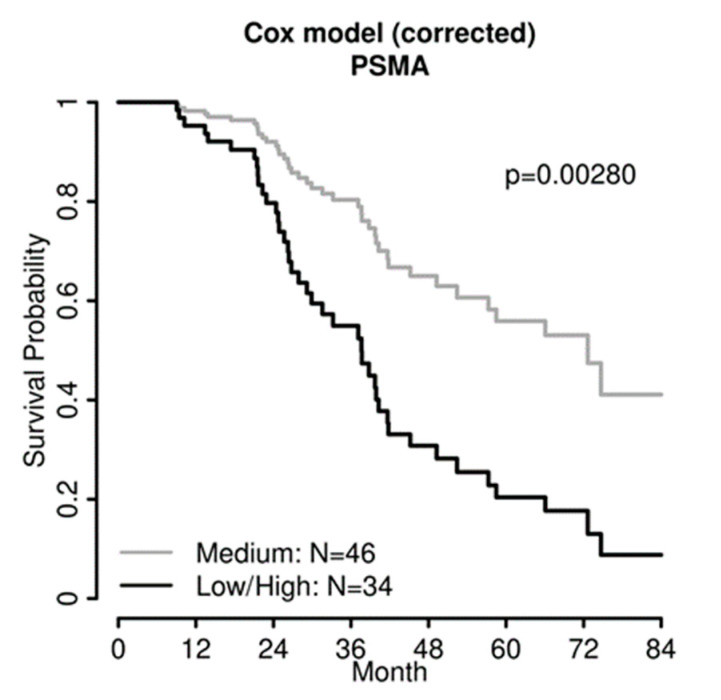
In multivariate analysis, PSMA expression was an independent marker for overall survival in 80 patients with advanced high-grade ovarian cancer, comparing two groups with “low/high” and “medium” PSMA expression. As these survival curves represent a multiple Cox regression model, no censored patients are indicated.

In univariate analyses, FIGO stage and residual disease were significantly associated with progression-free survival (Table 2). PSMA, dichotomized as described above, failed to reach statistical significance as a single factor (HR 1.61 (95% CI 0.98–2.64), *p* = 0.059). For overall survival, age, residual disease, and dichotomized PSMA expression (HR 2.08 (95% CI 1.12–3.87), *p* = 0.020) were significant prognostic factors (Table 3). In multiple Cox regression models, considering age, FIGO stage, and residual disease, “low/high” PSMA expression in the tumor-associated neovasculature was a significant and independent prognostic marker for progression-free (HR 2.24 (95% CI 1.32–3.82), *p* = 0.003) and overall survival (HR 2.73 (95% CI 1.41–5.29), *p* = 0.003; Figure 3).

A total of 29 of 34 (85%) of patients with “low/high” and 35 of 46 (76%) with “medium” PSMA expression received bevacizumab (*p* = 3.09). Bevacizumab was not associated with overall survival in models considering age, FIGO stage, residual tumor, and PSMA expression (HR 0.75 (95% CI 0.35–1.64), *p* = 0.476). In the subgroup of patients with bevacizumab therapy (*n* = 64), PSMA expression remained an independent significant prognostic marker for overall survival (HR 3.14 (95% CI 1.45–6.80), *p* = 0.004), including the same clinicopathological parameters as in Table 3.

## 4. Discussion

This is the first study showing a clinical relevance of PSMA expression in tumor-associated neovasculature in patients with HGSOC. Our study collective consists of a homogenous group of advanced stage high-grade serous cancers reflecting the majority of EOC patients. The present findings are in line with previous reports in solid cancers. PSMA expression was an independent adverse prognostic marker in oral squamous cell, thyroid, breast, hepatocellular, and renal carcinomas [2,3,4,5,6]. Interestingly, progression-free and overall survival was impaired in patients with absent, very low, and high PSMA as opposed to patients with medium PSMA expression ranging from 20 to 80% in our study. These thresholds were derived from a non-linear model of the overall survival hazard ratios using smooth splines and plotting the PSMA-dependent hazard ratios (HRs) against the PSMA expression. Cutoffs were set at the crossing of the HR estimate with the HR = 1 line, i.e., ≤10% and ≥90% (Figure 2B). This was in line with a Cox regression model using quartiles as stratification for PSMA expression (Figure 2A). In the literature, PSMA expression has been categorized in various ways. In thyroid cancers, Sollini et al. excluded cancers lacking PSMA, and compared cases exhibiting low (11–79%) and high (≥80%) expression levels [3]. Other studies combined cases without and with low (<50%) PSMA expression and compared them with cases with high (>50%) PSMA expression [2,4,5]. Except for one study in breast cancer, survival data are not provided separately for patients lacking PSMA [4]. The role of PSMA in endothelial cells of tumor-associated neovasculature has not been fully evaluated yet. PSMA is a membrane-bound glutamate carboxypeptidase that cleaves small extracellular matrix proteins such as laminin, which in turn activate endothelial cells and enhance angiogenesis [20]. A myriad of factors such as vascular endothelial growth factor (VEGF) and platelet derived growth factor (PDGF) are involved in angiogenesis [21]. Interestingly, VEGF and PDGF have been reported to possess prognostic relevance in patients with ovarian cancer [22]. We hypothesize that in cases with low PSMA expression, other angiogenesis-related factors may be up-regulated and thus impair prognosis.

Absent and very low PSMA expression was present in a subset of 18% ovarian cancer samples. This percentage lies in the previously reported range of 15 to 18% for absent PSMA in solid cancers [2,3,4,5,6]. Studies in ovarian cancer, however, are conflicting. Wernicke et al. detected PSMA expression in all 21 ovarian cancer samples with the majority exhibiting PSMA expression in more than 50% of vessels [13]. In contrast, Aide et al. did not find PSMA in 32 cases, using a commercially available PSMA antibody with intact positive controls [14]. Importantly, they did not delineate tumor-associated neovasculature using a CD31 antibody. They also did not comment on the size of their tumor samples. Tumor heterogeneity is a well-known phenomenon and can cause problems if tissue microarrays are used. We observed tumor-associated vessels predominantly in fibrovascular cores of cancer sections with papillary structure. Wernicke et al. examined ovarian cancer samples from multiple sites in 10 patients and reported that the percentage of PSMA positive vessels was either similar or higher in metastatic foci as compared to the primary ovarian tumor.

In the present study, PSMA was found in the vast majority of ovarian cancer samples, indicating that many patients may benefit from PSMA-directed imaging modalities. In advanced disease, PSMA-PET might improve detection rates of small peritoneal implants. Contrast-enhanced computed tomography has sensitivities and specificities of 25%–100% and 78%–100%, respectively, in detecting peritoneal carcinomatosis. These rates diminish significantly in lesions measuring less than 5 mm and in certain locations such as the root of mesentery, lesser omentum, left diaphragm, and serosal surfaces of the small bowel [23]. Novel imaging techniques might allow more accurate treatment planning and avoid futile surgeries if complete gross resection is not possible. These patients could directly proceed to neoadjuvant chemotherapy. The role of PSMA-PET in evaluating the distribution and extent of ovarian cancer is currently evaluated in clinical trials. In a prospective, single arm pilot study, 20 patients will have a PET using a novel, second-generation low-molecular weight PSMA, 18F-DCFPyL (2-(3-(1-carboxy-5-[(6-[18F]fluoro-pyridine-3-carbonyl)-amino]-pentyl)-ureido)-pentanedioic acid). Imaging results will be compared with conventional CT and intraoperative findings (NCT03811899). A second study aims to evaluate PSMA-based 18F-DCFPyL PET and magnetic resonance in 40 patients with ovarian cancer and a control group of healthy women (NCT03302156). In the future, PSMA-PET might also play a role in estimating treatment response or in the work-up of adnexal masses. If PSMA-PET was able to detect an early malignant process, preoperative staging and treatment planning could be improved. In pancreatic masses, the accuracy for PSMA-PET was 92.5% as compared to 72.5% for CT in diagnosing malignant lesions [24].

In the future, ovarian cancer patients might also benefit from innovative new techniques involving PSMA expression. Recently, PSMA has been detected in serum extracellular vesicles in patients with prostate cancer [25]. Finally, PSMA-directed treatment options could potentially play a role in the care of patients with ovarian cancer [12].

## 5. Conclusions

In conclusion, PSMA was present in the tumor-associated vasculature of the vast majority of high-grade serous ovarian cancer samples. PSMA expression dichotomized according to extreme value levels (“high/low” versus “medium” expression) were independent prognostic markers for progression-free and overall survival in patients with advanced disease. In the future, studies on PSMA-targeted diagnostic and therapeutic tools could be further evaluated in patients with ovarian cancer.

## Figures and Tables

**Table 1:** Clinicopathological parameters in 80 patients with high-grade serous ovarian cancer stratified by PSMA expression.

Parameter	“Low/High” PSMA *n* = 34	“Medium” PSMA *n* = 46	*p*-Value
Age (median, years)	62.5 IQR (52.4–70.3)	60.4 IQR (51.1–66.4)	0.65 ^1^
FIGO stage			0.83 ^2^
III	26	33	
IV	8	13	
Residual disease			1.00 ^2^
none	18	25	
any	16	21	
Vessel density			0.81 ^3^
low	3	2	
medium	11	15	
high	20	29	
Treatment response after chemotherapy ^4^ (complete and partial response)			0.34 ^2^
yes	27	42	
no	2	1	

^1^*t*-test. ^2^ Pearson′s chi-squared test. ^3^ Fisher′s exact test. ^4^ Data were available for 72 patients. IQR, interquartile range.

**Table 2:** Progression-free survival, univariate, and multivariate analyses.

*n* = 80	Progression-Free Survival			
64 Events	Univariate		Multivariate	
	HR (95% CI)	*p*-Value	HR (95% CI)	*p*-Value
Age	1.02 (0.10–1.04)	0.107	1.01 (0.99–1.04)	0.310
FIGO stage (IV > III)	2.18 (1.25–3.83)	0.006	2.07 (1.14–3.74)	0.016
Residual disease (any > none)	3.20 (1.89–5.43)	<0.001	3.37 (1.89–6.00)	<0.001
PSMA expression (“low/high” > “medium”)	1.61 (0.98–2.64)	0.059	2.24 (1.32–3.82)	0.003

**Table 3:** Overall survival, univariate, and multivariate analyses.

*n* = 80	Overall Survival			
42 Events	Univariate		Multivariate	
	HR (95% CI)	*p*-Value	HR (95% CI)	*p*-Value
Age	1.04 (1.01–1.07)	0.013	1.03 (1.00–1.06)	0.066
FIGO stage (IV > III)	1.16 (0.58–2.32)	0.681	1.23 (0.60–2.54)	0.570
Residual disease (any > none)	3.14 (1.67–5.87)	<0.001	3.71 (1.91–7.20)	<0.001
PSMA expression (“low/high” > “medium”)	2.08 (1.12–3.87)	0.020	2.73 (1.41–5.29)	0.003

## Data Availability

Data can be made available upon reasonable request.
